# Fiber Optic Sensor for Real-Time Sensing of Silica Scale Formation in Geothermal Water

**DOI:** 10.1038/s41598-017-03530-1

**Published:** 2017-06-13

**Authors:** Takuya Okazaki, Tatsuya Orii, Akira Ueda, Akiko Ozawa, Hideki Kuramitz

**Affiliations:** 1 Department of Environmental Biology and Chemistry, Graduate School of Science and Engineering for Research, University of Toyama, Gofuku 3190, Toyama, 930-8555 Japan; 2 Natural Resources, Environment and Energy Engineering Division, Mitsubishi Materials Techno Corporation, Kudankita 1-14-16, Chiyoda-ku, Tokyo 102-8205 Japan

## Abstract

We present a novel fiber optic sensor for real-time sensing of silica scale formation in geothermal water. The sensor is fabricated by removing the cladding of a multimode fiber to expose the core to detect the scale-formation-induced refractive index change. A simple experimental setup was constructed to measure the transmittance response using white light as a source and a spectroscopy detector. A field test was performed on geothermal water containing 980 mg/L dissolved silica at 93 °C in Sumikawa Geothermal Power Plant, Japan. The transmittance response of the fiber sensor decreased due to the formation of silica scale on the fiber core from geothermal water. An application of this sensor in the evaluation of scale inhibitors was demonstrated. In geothermal water containing a pH modifier, the change of transmittance response decreased with pH decrease. The effectiveness of a polyelectrolyte inhibitor in prevention of silica scale formation was easily detectable using the fiber sensor in geothermal water.

## Introduction

One of the serious problems with the use of geothermal water is the scale formation of inorganic salts such as calcium carbonate (CaCO_3_), amorphous silica, or calcium sulfate. Changes in temperature and pressure influence the equilibrium by inducing scale formation on the wall surface of wells, flow lines, valves, turbines, and separators^1^. Such scale formation results in a gradual decrease water flow rate and heat-exchange efficiency of a given system. This results in exorbitant operating costs for maintenance, replacement, and removal of scale from equipment. The estimated cost spent to tackle scale formation problem in the industrialized world is estimated to be 26,850 million USD^2, 3^.

Numerous techniques have been reported for prevention of CaCO_3_ scale formation, such as the addition of chemical inhibitors including polyelectrolytes, organophosphates, ethylenediaminetetraacetate, metal ions, and nanoparticles and other techniques involving surface modification of equipment, pH modification, and ultrasonic or electromagnetic irradiation^2–11^. On the other hand, although silica scale is observed in geothermal plants in equal or greater frequencies than CaCO_3_ scale, there are significantly fewer reports in the literature concerning silica scale prevention techniques; these techniques include the addition of polyelectrolytes, borates, chelating reagents, and dendrimers, and pH modification^11–22^. The applications of these methods in geothermal fields are also limited. One of the reasons is that the effectiveness of these techniques for silica scale prevention has not been identified due to the complex mechanism of silica scaling^12, 23, 24^. In addition, it has been reported that silica scaling is exacerbated by the addition of a slightly excess amount of inhibitors, which causes the acceleration of agglomeration or precipitation of silica^17, 25, 26^. Therefore, the effectiveness of the silica scale prevention techniques should be carefully evaluated to understand the behavior of silica scaling and to recognize potential techniques that could further contribute to overcoming this problem.

Table 1 shows the methods for monitoring silica scale formation reported in literature on scale prevention or control. Most of these methods can be classified as weighing the deposited scale, chemical analysis of soluble silicate, and flow rate monitoring in test equipment. The weighing methods provide the amount of scale precipitated on experimental equipment^12, 14, 25, 27, 28^. However, such methods require a long deposition time to detect the change in the scale amount obtained by a balance. The chemical analysis of soluble silicate with molybdate colorimetry is useful in the investigation of the polymerization behavior of silicates^13, 15–17, 21^. Nevertheless, this method is an indirect evaluation of scale formation, time consuming, and complicated for continuous monitoring. Flow rate monitoring methods enable a direct evaluation of the effect of scaling on flow properties^18, 19^. Such methods can easily allow continuous monitoring of the flow rate and hence the scale formation in equipment. However, they require design and fabrication of experimental flow systems and it takes a long time (a few days to a week) for scale formation to affect the flow rate. Even though other techniques, such as turbidity measurement, gamma ray, and ultrasonic, have been suggested over the years to monitor the scaling phenomena, the problem still exists, and thus creates a potential impact on our economy^29–31^.

**Table 1:** Examples of methods of monitoring silica scale formation in the literature on scale prevention or control.

Monitoring methods	Situation	Scale	Scale inhibitor or controller	Measurement period	Reference
Weighing the scale collected in spools	Laboratory and geothermal field	Silica	Mixed chemical inhibitor	48 h	25, 27
Measurement of monomeric silica in water by molybdate colorimetry	Laboratory	Aluminum silicate	pH modification Chelating reagent	4 h	13
Weighing the scale collected in spools	Laboratory and geothermal field	Aluminum silicate	Chelating reagent	48 h	14
Monitoring the flow rate through a column packed with alumina beads	Geothermal field	Silica	pH modification	150 h	18
Monitoring the flow rate through a fluidized-bed reactor	Geothermal field	Silica	pH modification Chemical impurities	48 h	19
pH measurement	Geothermal field	Silica	pH modification with biochemical reactor	30 days	20
Measurement of monomeric silica in water by molybdate colorimetry	Geothermal field	Silica	pH modification	200 days	21
Weighing the scale deposited on stainless steel rods	Geothermal field	Metal silicate	Commercial inhibitors	24 h	28
Measurement of silica in water by molybdate colorimetry	Cooling water	Silica	Dendrimers	72 h	16, 17
Weighing the scale collected in column	Cooling water	Silica	Polyelectrolyte	10 days	12
Measurement of monomeric silica in water	Reverse osmosis treatment	Silica	Chelating reagent	9 h	15

Over the past two decades, there has been a clear trend toward the use of fiber optics in the detection of chemicals^32–36^. Several groups have reported the fiber optic method for monitoring CaCO_3_ precipitation by using an exposed core fiber and monochromatic laser light^37–40^. In our previous work, we proposed an optical fiber sensor that uses an exposed core fiber, white light, and a spectroscopy detector for monitoring CaCO_3_ scale formation in geothermal water^41^. The detection principle of this sensor was based on the percentage of total internal reflection within the fiber optic core, which is affected by the high refractive index of CaCO_3_ scale formed on the surface of an exposed core. The advantages of this sensor include high sensitivity, real-time remote monitoring, heat resistance, small size, ease of handling, and cost effectiveness. Moreover, the design and fabrication of a specific experimental system mentioned above are not required for geothermal scaling monitoring.

In this study, a novel fiber optic sensor for silica scale formation was developed under experimental conditions. The field test was conducted at Sumikawa Geothermal Power Plant to analyze the performance of the sensor. The novelty of this study is the development of a sensor for monitoring silica scale formation in geothermal water in real time. To the best of our knowledge, this is the first reported case of a sensor that can evaluate the effectiveness of scale inhibitor. Real-time monitoring is necessary because the reaction of scale formation in geothermal water is unstoppable and ever changing.

## Results

### Laboratory study

**Figure 1:**
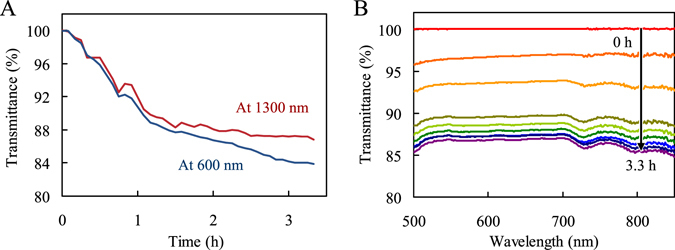
(**A**) Transmittance response monitored by the fiber sensor with 8 cm exposed core length at 600 nm (blue line) and 1300 nm (red line) as a function of time after immersion in a solution containing 1000 mg/L silicate (as SiO_2_). (**B**) Spectral changes with time from 500 to 850 nm with the fiber sensor under the same conditions. Curves from top to bottom represent increase in time.

Transmittance responses were measured at wavelengths ranging from 500 to 1700 nm after the fiber sensor was immersed in a silicate solution (1000 mg/L as SiO_2_) at 90 °C. The transmittance decreased over time, as shown in Fig. 1(A). Broad lines were observed in the range from 500 to 850 nm, as indicated in Fig. 1(B). The transmittance response of the sensor showed slightly better sensitivity in the near-infrared region than in the visible region. This tendency is similar to the results of our previous measurement for CaCO_3_ scale, which has a higher refractive index than the quartz fiber core^41^. The result indicates that the sensor is able to detect not only CaCO_3_ but also silica scale formed in geothermal water.

### Field study

**Figure 2:**
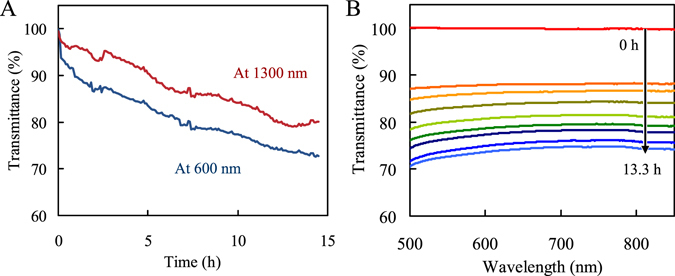
(**A**) Transmittance response monitored by the fiber sensor with 24 cm exposed core length at 600 nm (blue line) and 1300 nm (red line) as a function of time after immersion in geothermal water at Sumikawa Geothermal Power Plant. (**B**) Spectral changes with time from 500 to 850 nm with the fiber sensor under the same conditions. Curves from top to bottom represent increase in time.

To evaluate the effectiveness of the fiber sensor in monitoring silica scale formation, a field test was performed on geothermal water containing 980 mg/L dissolved silica as SiO_2_ at 93 °C in Sumikawa Geothermal Power Plant, Japan. As shown in Fig. 2(A), the transmittance response decreased due to scale formation in geothermal water. The sensor detection region at visible wavelengths showed better sensitivity than at near-infrared wavelengths. This result is in disagreement with the laboratory result. Philip-Chandy *et al*. reported that transmitted light decreases as a function of increasing refractive index of the cladding when the cladding has a lower refractive index than the fiber core^42^. The higher sensitivity at visible wavelengths could be explained by wavelength dependence of the refractive index of silica formed on the quartz fiber core: the refractive index of silica increases as the wavelength decreases^43^. Moreover, it seems that the difference in results between laboratory and field tests could be related to the slight difference in purity between pure SiO_2_ formed in the laboratory test and silica scale precipitated in geothermal water in the field test^12^. The transmittance spectrum in the field test showed broad lines in agreement with the laboratory result. After the field test, the scale was analyzed by XRD measurement, which showed only the peak of silica.

### Effect of exposed core length

**Figure 3:**
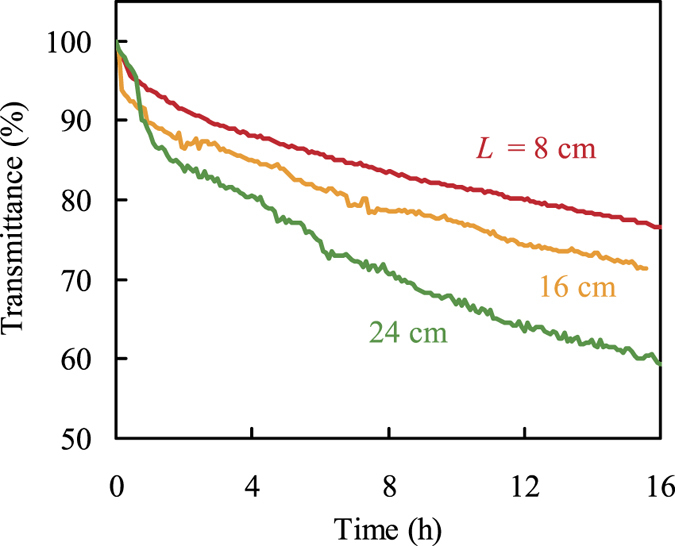
Effect of the exposed core length on the transmittance response monitored by the fiber optic sensor as a function of time after exposure to geothermal water in Sumikawa Geothermal plant, Japan. Wavelength: 600 nm; exposed core length: 8 cm (red line), 16 cm (yellow line), and 24 cm (green line).

The effect of exposed core length was examined by varying the length of exposed fiber core in geothermal water, as shown in Fig. 3. The transmittance value obtained from the sensor increased with increase in the exposed fiber core length. In this figure, the tendency of decreasing transmittance in all results is divided into two steps, the initial rapid change and the subsequent gradual change. It seems that the first step is due to precipitation of silica scale on the fiber core and the second step is caused by growth of silica covering the fiber core. The slope of the transmittance decline in the second step also depended on the exposed fiber core length. This might be related to the total amount of scale precipitated on the fiber core.

### Evaluation of pH modifier

**Figure 4:**
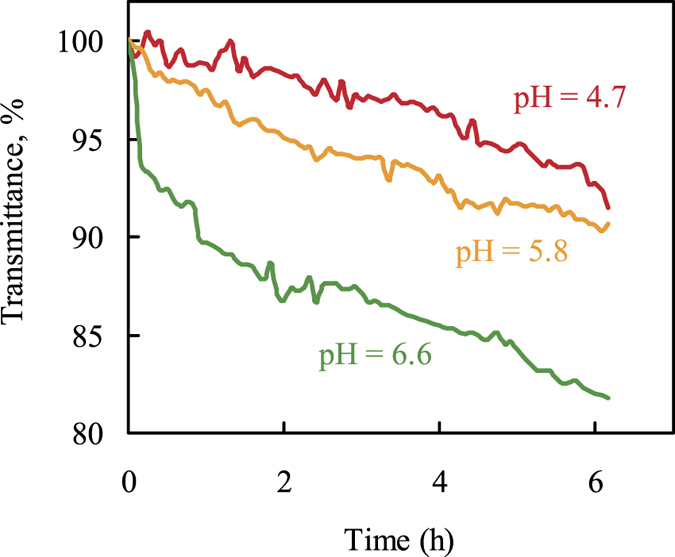
Effect of the pH modifier on the scale formation monitored by the fiber optic sensor. Exposed core length: 24 cm; wavelength: 600 nm; pH values: 4.7 (100 mg/L, red line), 5.8 (10 mg/L, yellow line), and 6.6 (0 mg/L, green line).

The use of a pH modifier as a silica scale inhibitor was examined in geothermal water at Sumikawa Geothermal Power Plant. In these tests, the exposed core length of 24 cm was used, which has the highest sensitivity as in the previous results. Changes in transmittance with time at different concentrations of the pH modifier in geothermal water are shown in Fig. 4. The change in transmittance decreased with decreasing pH in geothermal water. It is well known that silica scale deposition is affected by the solution pH because the solubility of silica scale increases with a decrease in pH over a pH range of 4–9^13^. Our results agreed with this known tendency. It is shown that the sensor can clearly measure the effect of pH change on silica scale formation within 6 h.

### Evaluation of polycarboxylic acid inhibitor

**Figure 5:**
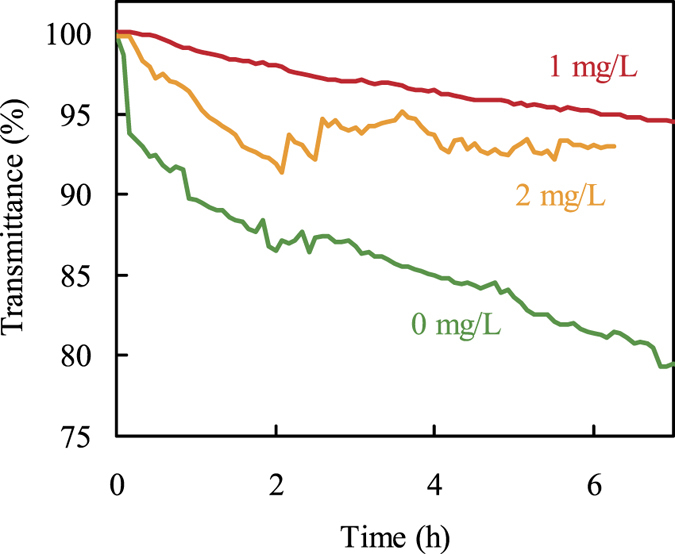
Effect of the polycarboxylic acid inhibitor on the scale formation monitored by the fiber optic sensor. Exposed core length: 24 cm; wavelength: 600 nm; inhibitor concentration: 1 mg/L (red line), 2 mg/L (yellow line), and 0 mg/L (green line).

Figure 5 shows the transmittance response of the fiber optic sensor in geothermal water containing a polycarboxylic acid inhibitor. The transmittance change at an inhibitor concentration of 1 mg/L was clearly smaller than that of 0 mg/L. This result shows that the sensor could detect the effect of the scale inhibitor in geothermal water within a few hours. On the other hand, the rate of transmittance decrease at an inhibitor concentration of 2 mg/L was larger than that at 1 mg/L. Gallup reported that higher concentrations of an organic inhibitor at the ppm level enhanced scale formation due to flocculation or agglomeration of silica^25^. In this experiment, the effectiveness of the organic inhibitor for the inhibition of scale formation reduced at a concentration of 2 mg/L. The key finding from the experiment is that the fiber optic sensor could easily detect the small differences in the inhibitor effectiveness during a short time (less than 7 h) in geothermal field. This rapid and continuous observation of scale formation will contribute to the development of techniques for silica scale prevention in geothermal water.

## Discussion

To measure the amount of scale formed on the fiber sensor, the scale thickness was measured by vertical scanning interferometry using quartz plates immersed in geothermal water, as shown in Table 2. The scale thickness linearly increased with the deposition time in geothermal water, and the average growth rate of the scale was 15.5 nm/h. By using this value, the rates of increase in the volume of scale formed on the exposed fiber core were calculated. For a 200-μm-diameter fiber with lengths of 8, 16, and 24 cm, the scale volume growth rates are 0.80 × 10^−3^, 1.59 × 10^−3^, and 2.39 × 10^−3^ mm^3^/h, respectively. The distances between these values are in close agreement with absorbance increment as a function of time, which is calculated from the transmittance responses in Fig. 3. From these results, the relationship between the absorbance of the fiber optic sensor and the weight of scale formed on the fiber core can be represented as

$$A=-\mathrm{log}(I/{I}_{0})=9.64m+{A}_{{\rm{i}}}$$

where *A* is the sensor absorbance, and *I*_0_ and *I* are the light intensity before and after scale formation, respectively, *m* is the weight of scale formed on the fiber core (calculated using quartz density = 2.635)^44^, and *A*_i_ is the small change in absorbance in the first step mentioned above. From the equation, this sensor is sensitive to the weight of scale formed on the fiber core and its sensitivity can be increased by increasing the exposed fiber core length, as this increases the total amount of scale. Therefore, the sensitivity and working time of the fiber sensor for monitoring silica scale in geothermal water can be easily controlled by the length of the exposed fiber core. Moreover, the results showed that this sensor can detect a continuous change of scale weight at the 1 μg level, whereas the typical weighing technique for silica scale monitoring uses balances with a precision of 0.1 mg.

**Table 2:** Scale thickness on quartz plates in geothermal water (n = 3).

Deposition time (h)	Scale thickness (μm)
22.8	0.36 ± 0.01
46.8	0.63 ± 0.02
70.8	1.14 ± 0.03
118.8	2.08 ± 0.02
166.8	2.45 ± 0.27

## Methods

### Chemical reagents and materials

Sodium metasilicate nonahydrate and sulfuric acid was purchased from Wako Pure Chemicals Industries (Japan). The pH modifier consisting of maleic acid and polyacrylate was obtained from Techno Office Japan. The polycarboxylic acid inhibitor was supplied by BWA Water Additives Japan Limited.

A step-index multimode optical fiber (FT200EMT; Thorlabs, USA) with a 200-μm-diameter fused silica core was used. The fiber core has a refractive index of 1.451, and it is surrounded by TEQS^TM^ polymer cladding with a refractive index of 1.392 at 1020 nm. The fiber cladding was carefully removed from the middle of the fiber by rubbing to expose the fiber core.

A white light source (ELI-050J-OPT3077; Mitsubishi Rayon, Japan) attached with a halogen lamp (JCR12V-50WGAL; Ushio, Japan) was used to perform light coupling between the white light source and a spectroscopy detector (SA-100VRD VIS-NIR; Lambda Vision, Japan) through the fiber sensor. Sensor transmittance values were acquired by recording the light intensity through the fiber before exposure to scale formation (*I*_0_) and the intensity after exposure to scale formation as time proceeds (*I*). Sensor transmittance in the fiber sensor was defined as T (%) = (*I*/*I*_0_) × 100. It is confirmed that the variation of the transmittance obtained from the system that utilized an unprocessed fiber optic was less than 1% over a time span of 10 h. A stabilized power supply was used during the field experiments. However, if a stable transmittance cannot be obtained, then an optical switch of 1 × 4 for multipoint monitoring was applied. The unprocessed fiber is connected to one of the ports at the optical switch and is used as a reference for optical intensity.

### Laboratory study

The optical fiber was placed on a watch glass by applying tension to both ends of the fiber with the middle portion immersed in the solution at some distance from the bottom of the watch glass. The silica scale solution was prepared by dissolving sodium metasilicate (1000 mg/L) as SiO_2_ in water at 90 °C. After the solution was neutralized to pH 8 by adding sulfuric acid, the exposed core of the fiber fixed on the watch glass was immediately immersed in the solution.

### Field study

A field study was carried out in a geothermal water environment at Sumikawa Geothermal Power Plant, Japan (50,000 kW). Two-phase flow from SC-4 production wells is flowed into a 50 L simplified separator and separated into steam and water. The chemical parameters of the geothermal water are shown in Table 3. Atomic absorption spectroscopy was used to determine the concentration of Na and K, whereas Ca, Mg, Mn, Al, and total Fe concentrations were determined using inductively coupled plasma atomic emission spectrometry. Gravimetry was used to measure SiO_2_ and SO_4_ concentrations. Phenanthroline spectrophotometry was used to measure the Fe(III) concentration, and Fe(II) concentration was calculated by subtracting the Fe(III) concentration from the total Fe concentration. HCO_3_ and Cl^−^ concentrations were measured using titration method. Two different electrodes were used to measure pH and electrical conductivity (EC). Geothermal water was flown at a rate of 1050 mL/min over the fiber sensor fixed in a bucket with tension applied to both ends. In inhibitor evaluation, the inhibitor solution was mixed with geothermal water by flowing it through a T-tube connected with the tube flowing geothermal water using a pump. The inhibitor concentration was calculated from the flow rate of the inhibitor solution and geothermal water.

**Table 3:** Chemical composition of groundwater sampled in Sumikawa Geothermal Power Plant, Japan.

Temperature (°C)	93.3	SO_4_ (mg/L)	99
pH	6.48	HCO_3_ (mg/L)	15
EC (mS/m)	414	SiO_2_ (mg/L)	980
Na (mg/L)	680	Mn (mg/L)	0.07
K (mg/L)	110	Fe(II) (mg/L)	< 0.1
Ca (mg/L)	16.0	Fe(III) (mg/L)	0.04
Mg (mg/L)	0.05	Al (mg/L)	1.0
Cl (mg/L)	1100		

### Measurement of scale thickness

For measuring of the thickness of scale formed on the quartz fiber core in geothermal water, quartz plates with dimensions of 10 mm × 10 mm × 2 mm were prepared. A small gold leaf was adhered to a part of the quartz plates. The plates were immersed into the bucket through which geothermal water flowed at 1050 mL/min. After a certain time, the quartz plate was taken out from the water. After removing the gold leaf from the quartz plates using an ultrasonic bath, the difference in thickness between the surface of scale formed and the bare surface of the quartz plates was measured by vertical scanning interferometry (MM5500; Ryoka Systems, Japan).
